# Valorization of Spent *Escherichia coli* Media Using Green Microalgae *Chlamydomonas reinhardtii* and Feedstock Production

**DOI:** 10.3389/fmicb.2017.01026

**Published:** 2017-06-07

**Authors:** Jian-Guo Zhang, Fang Zhang, Kiran Thakur, Fei Hu, Zhao-Jun Wei

**Affiliations:** School of Food Science and Engineering, Hefei University of TechnologyHefei, China

**Keywords:** anaerobic broth, acetic acid, nutrients removal, microalgae, *Chlamydomonas reinhardtii*

## Abstract

The coupling of *Chlamydomonas reinhardtii* biomass production for nutrients removal of *Escherichia coli* anaerobic broth (EAB) is thought to be an economically feasible option for the cultivation of microalgae. The feasibility of growing microalgae in using EAB high in nutrients for the production of more biomass was examined. EAB comprised of nutrient-abundant eﬄuents, which can be used to produce microalgae biomass and remove environment pollutant simultaneously. In this study, *C. reinhardtii* 21gr (cc1690) was cultivated in different diluted *E. coli* anaerobic broth supplemented with trace elements under mixotrophic and heterotrophic conditions. The results showed that *C*. *reinhardtii* grown in 1×, 1/2×, 1/5× and 1/10×*E. coli* anaerobic broth under mixotrophic conditions exhibited specific growth rates of 2.71, 2.68, 1.45, and 1.13 day^-1^, and biomass production of 201.9, 184.2, 175.5, and 163.8 mg L^-1^, respectively. Under heterotrophic conditions, the specific growth rates were 1.80, 1.86, 1.75, and 1.02 day^-1^, and biomass production were 45.6, 29.4, 15.8, and 12.1 mg L^-1^, respectively. The removal efficiency of chemical oxygen demand, total-nitrogen and total-phosphorus from 1×*E. coli* anaerobic broth was 21.51, 22.41, and 15.53%. Moreover, the dry biomass had relatively high carbohydrate (44.3%) and lipid content (18.7%). Therefore, this study provides an environmentally sustainable as well economical method for biomass production in promising model microalgae and subsequently paves the way for industrial use.

## Introduction

Research on microalgae for producing biofuels has mainly focused on enhancing microalgal biomass yield or lipid content, but biofuels production based on microalgae remains uneconomical because of the high cost of feedstock. Lately, microalgae are gaining increasing attention of the researchers attracted due to its potential to serve as feedstock for biofuels. Wastewater is considered an ideal niche for cultivation of microalgae with an aim to obtain microalgal feedstock for biofuel use. Therefore, microalgae-based wastewater treatment has been considered as an alternative strategy for various types of wastewater treatment due to their capability of consuming the contaminants (associated removal of inorganic nutrients like nitrogen, phosphorous, heavy metals etc.) and producing biomass (Aravantinou et al., 2013). The obtained microalgal biomass can find its way in the downstream processes, including biofuels (bioethanol, biodiesel, biomethane and biohydrogen), animal feeds, and other high value products such as astaxanthin and carotenoid (Ding et al., 2016).

Microalgae biomass can be refined into a variety of industrial productions such as biofuels, pigments, cosmetic, nutritious food and animal feed (Yen et al., 2013). However, industrial production of microalgae still requires extensive basic and applied work because of several problems yet to be solved; high cost is considered as a technical obstacle. Photoautotrophic growth in photo bioreactor is a traditional method that has been used for microalgae cultivation, and currently it is extensively applied in bench- (Xie et al., 2013), pilot- (Chen et al., 2011) and large-scale (Arad and Richmond, 2004) system. Compared to autotrophic cultivation, heterotrophic or mixotrophic system pose several advantages such as higher biomass density, higher growth rates and higher lipid yields, thus improve economic competitiveness of microalgae products (Ceron Garcia et al., 2006; Moon et al., 2013). At present, many organic substrates had been reported as effective carbon resource during heterotrophic microalgae growth (Ceron Garcia et al., 2006; Morales-Sanchez et al., 2013). However, pure substrates (i.e., glucose, sucrose, glycerol and ethanol) may not support substantial heterotrophic growth of microalgae for the higher economic cost. Among the microalgae, *Chlamydomonas reinhardtii* is a widely distributed microalgae species, and it serves as a model organism for studying the regulation of metabolic pathways (Moon et al., 2013).

The last decade has witnessed the rapid growth in the biofuels industry which led to massive pressure on food and animal feed supplies and agricultural land uses. In order to the biofuels industry to sustain and continue to grow, new non-food or non-feed biomass feedstock must be explored and developed (Kong et al., 2010). Therefore, there should be shift toward cost effective strategies for microalgae cultivation. Organic eﬄuents are considered as the most promising alternatives for low cost of microalgae cultivation. Among the organic eﬄuents, acetate is a common by-product of anaerobic digestion and can be found in most fermentation eﬄuents such as hydrogen fermentation broth, methane fermentation mixture and landfills leachate. Microalgae can easily convert acetate into acetyl-CoA which is the main precursor for carbon compounds synthesis (Ramanan et al., 2013). Thus, coupling of the eﬄuents treatment to microalgae production cannot only decrease the feedstock’s cost but also benefit environment protection (Lau et al., 1996).

Previous report suggested that municipal, agricultural and industrial wastewater showed great potential for microalgal growth (Chiu et al., 2015). Though, the combined environmental benefits of wastewater treatment and microalgal CO_2_ fixation provide economic incentives to society (Wang et al., 2008; Qi et al., 2017). There are many studies on microalgae cultivation in wastewater under laboratory conditions and in higher scale, (Álvarez-Díaz et al., 2017). The mechanisms of CO_2_ utilization by *C. reinhardtii* in waste water are still poorly understood. On the other hand, recent reports have reported that culturing microalgae in mixotrophic mode with diluted primary piggery wastewater provided an effective method, besides its nutrient-rich composition, high concentrations of urea, ammonia, organic carbons and pesticides, high turbidity and unbalanced C:N:P ratios are also present which affect the growth of algae (Wang et al., 2012; Qi et al., 2017). Thus, our study has shifted the traditional trend by utilizing the *Escherichia coli* nutrient broth which is a novel and interesting approach.

The results of biomass accumulation and nutrients removal efficiency depend on the species of microalgae; the results would change if the species change. *C. reinhardtii* is a typical species of green algae which served as model organism for studying metabolic pathways (Boyle and Morgan, 2009) and can also be exploited in various experimentations. Moreover, this microalgal strain has the potential to grow in diverse conditions and to remove nutrients efficiently (Wu et al., 2012; Ding et al., 2016). The primary objective of this study was to develop creative methods to produce an economically and environmentally sustainable feedstock from algae grown on nutrients from bacteria spent media. This is expected to create a win–win situation where algal biomass production is improved through the elimination of phosphorus and nitrogen, while renewable energy can be generated using the spent media from the algae to produce biofuels. In this study, only one species of algae was screened and tested. Till date, there is no report of microalgae cultivation in bacterial growth media. In order to considerate the nutrient efficiency of *E. coli* anaerobic broth, the present work was executed. We intended to develop an economical and nutrient rich medium for microalgae cultivation.

In this study, *C. reinhardtii* 21gr (cc1690), the model eukaryotic green algae, was employed to assimilate the soluble metabolites from anaerobic broth of *E. coli* DH5α grown on glucose. The biomass, lipid production and nutrients removal were simultaneously investigated.

## Materials and Methods

### Preparation of *E. coli* Anaerobic Broth

*Escherichia coli* DH5α was purchased from TAKARA Biotechnology (Dalian) Co., Ltd and a modified minimal-medium was used for its anaerobic fermentation according to early description (Arora, 2012). The minimal-medium contained (g L^-1^): Glucose, 10.0; (NH_4_)_2_SO_4_, 2.0; K_2_HPO_4_, 1.0; NaH_2_PO_4_, 0.9; MgSO_4_7H_2_O, 0.2. The *E. coli* DH5α inoculum was pre-grown in Luria–Bertani (LB) medium under aerobic conditions (Su et al., 2015), washed with distilled water and recovered to initial volume with the minimal-medium. Subsequently, 600 mL of minimal-medium was inoculated with 400 mL of *E. coli* DH5α inoculum and incubated statically at 37°C for 72 h.

### Cultivation of *C. reinhardtii* in *E. coli* Anaerobic Broth

The *E. coli* anaerobic broth was centrifuged at 10,000 × *g* for 5 min. The harvested supernatant was neutralized with tris(hydroxymethyl)aminomethane and diluted to 1/2×, 1/5× and 1/10× with distilled water. To optimize nutrient source, all diluted and original broth was amended with appropriate trace element based on Tris-Acetate-Phosphate (TAP) medium (Liu et al., 2013). This supplemented broth was autoclaved and named as EAB (*E. coli* Anaerobic Broth).

Wild-type *C. reinhardtii* strain 21gr (cc1690) was obtained from the Chlamydomonas Resource Center^1^. The *C. reinhardtii* inoculum was cultured in TAP medium under continuous illumination (700 lux). Then, 5 mL of the microalgae inoculum was inoculated into 100 mL of EAB at four different dilutions (1×, 1/2×, 1/5×, and 1/10×). TAP medium was used as positive control. These cultures were grown at 25°C under continuous illumination (1500 lux) and shaken at 100 r/min on an orbital shaker.

### Measurement of *E. coli* Anaerobic Broth

*Escherichia coli* cell densities were verified by determining colony forming units. Reducing sugar was estimated calorimetrically with 3,5-dinitrosalicylic acid using glucose as standard (Kahraman and Yesilada, 2003). The measurement of acetate acid content was based on the procedure of Patel et al. (2008).

### Analytical Methods

The microalgae cell concentration was determined by measuring the optical density of algal culture at 680 nm. The microalgae biomass production (X; mg/L) was measured as dry weight and 100 or 500 mL of sample was collected and centrifuged at 12,000 × *g*, and 25°C for 5 min. Following that, the supernatant was removed for other analysis and the cell paste was dried at 105°C in an oven for 24 h and weighed.

The specific growth rate μ (day^-1^) in the exponential phase of microalgae growth was calculated by Eq. (1) (Zhu et al., 2016):

(1) $$\mu ({\text{day}}^{-1})=\left(\ln X_{\text{t}}-\ln X_{0}\right)/t$$

where, *X*_0_ refers to initial *X*-value and *X*_t_ signifies *X*-value after *t* days.

The contents of chemical oxygen demand (COD), total-nitrogen (TN) and total-phosphorus (TP) were evaluated following the description of Wang et al. (2015). Carbohydrate content was measured according to methods described by Gupta et al. (2016). Lipid content and fatty acid composition were determined using the method of Iwai et al. (2015).

## Results

A previous research showed that the microalgae *C. reinhardtii* could achieve the maximum biomass growth rate of 2.0 g L^-1^day^-1^ when it grew in the municipal wastewater in 10 days period. Meanwhile, nitrogen and phosphorus were removed effectively by the concentrationof 55.8 and 17.4 mg L^-1^day^-1^, respectively (Kong et al., 2010).

Another study has also indicated the potential of microalgae for biomass production and palm oil mill eﬄuent (POME) nutrients removal (Ding et al., 2016). The volatile fatty acids (VFAs)were reported as an inexpensive alternative carbon source for maximizing lipid production in mixotrophic cultivation of *C. reinhardtii* with an aim to determine the optimal conditions for growth and biodiesel production (Moon et al., 2013). Their results have also indicated that *C. reinhardtii* is not suitable for the heterotrophic cultivation. However, mixotrophic cultivation of *C. reinhardtii* led to increased biomass and lipid production (Moon et al., 2013).

Though, there have been extensive studies about wastewater and swine waste as an important niches for microalgae cultivation, besides their nutrient-rich compostion, high concentrations of urea, ammonia, organic carbons and pesticides, high turbidity and unbalanced C:N:P ratios are also present which possibly can affect the growth of algae. Moreoevr, the complex composition can lead slower growth rate. On the other hand, *E. coli* is very commonly used bacteria and its growth mdeia is very selective for cultivation of microalage as it contains optimal growth factors to achieve the targeted biomass in short time span as the nutrients are converted into simpler form by *E. coli* with enhanced bioavailability. This alternative can be considered as simpler, rapid, real-time and cost effective approach for microalgae gowth kinetics. Further, it can possibly shorten the lag growth phase and the alage can make up to log phase in shorter periods.

### Characteristics of *E. coli* Anaerobic Broth (EAB)

*Escherichia coli* DH5α is often chosen as host in many bioprocesses. Acetate is a major by-product during *E. coli* fermentation because it can be accumulated by ackA-pta pathway in exponential phase (Dittrich et al., 2005) and by poxB pathway in stationary phase (Abdel-Hamid et al., 2001).

**FIGURE 1:**
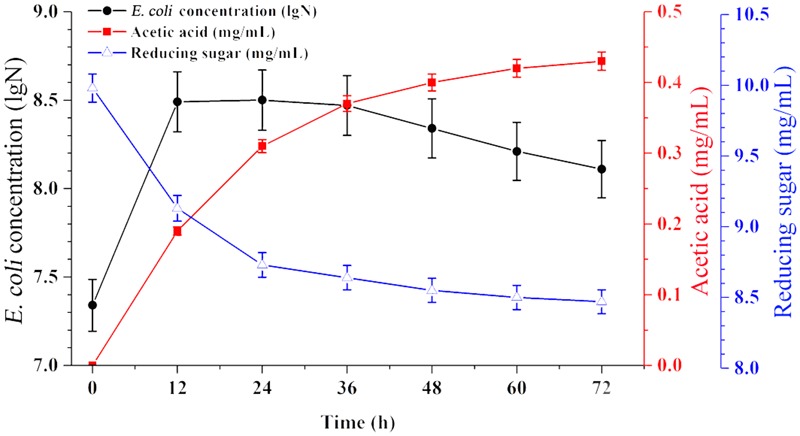
Broth characteristics of *E. coli* grown on glucose anaerobically. Data are represented as the means ± SD (*n* = 3).

Time-course profiles of EAB characteristics are shown in **Figure 1**. The results showed that the concentration of acetic acid increased from 0 to 0.43 mg/mL when the concentration of reducing sugar decreased from 10.0 to 8.47 mg/mL during 72 h of anaerobic fermentation. *E. coli* is facultative anaerobe, it’s flexible to switch between aerobic and anaerobic growth regime, based on the culture conditions. In this experiment, part of *E. coli* cells in the top broth may have access to oxygen and thus are able to perform aerobic respiration, however, anaerobic respiration could define the growth regime for other cells survived in the middle and bottom part of flask. This respiration state is similar with the cells in the industrial bioreactors.

As a typical species, *E. coli* can undergo four growth phases (lag, exponential, stationary, and lysis). In this case, no significant lag phase appeared since a large inoculum size was used (40%). The exponential phase persisted for 12 h, and most of the production of acetic acid and consumption of glucose have mainly taken place in this period. Subsequently, the EAB was collected and used as substrate for microalgae growth in the next experiments.

### Microalgae Growth in EAB

**FIGURE 2:**
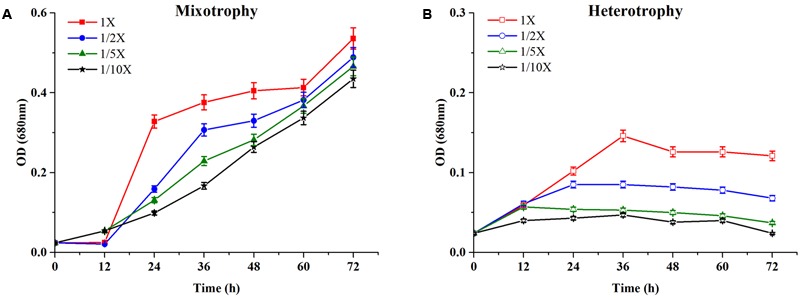
Growth profiles of *C. reinhardtii* under mixotrophic **(A)** and heterotrophic **(B)** conditions in four dilutions of EAB. Data are represented as the means ± SD (*n* = 3).

The growth characteristics of *C. reinhardtii* under mixotrophic and heterotrophic conditions were investigated. **Figure 2A** represented the growth profile of *C. reinhardtii* under mixotrophic condition in four media with different dilutions of EAB.

**FIGURE 3:**
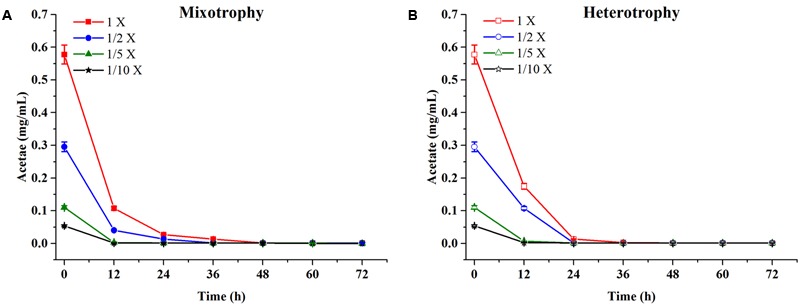
Acetate content profiles of *C. reinhardtii* under mixotrophic **(A)** and heterotrophic **(B)** conditions in four dilutions of EAB. Data are represented as the means ± SD (*n* = 3).

*Chlamydomonas reinhardtii* displayed significant diauxic growth in the media with EAB ratios of 1×, 1/2×. After 12 h of lag phase, 1× and 1/2× EAB culture entered exponential phase utilizing acetate as carbon source. Acetate was exhausted at 48 h and 36 h in 1× and 1/2× EAB, respectively (**Figure 3A**) and the stationary phase persisted for 12 h. Subsequently, they grew dramatically under autotrophic conditions following similar growth rate. *C. reinhardtii* did not illustrate diauxic growth when it grew in the media with EAB ratios of 1/5× and 1/10×. It might be due to the fact the acetate in their media was too rare and they took autotrophic growth initially. This hypothesis can be proved by their growth curve without significant lag phase compared to microalgae grown in 1× and 1/2× EAB.

As listed in **Table 1**, *C. reinhardtii* grown in 1×, 1/2×, 1/5×, and 1/10× EAB achieved different biomass productions and specific growth rates because the acetate concentration of the four media were different. Our results suggested that higher acetate concentrations contributed to higher biomass production and higher specific growth rates. *C. reinhardtii* that grew in the 1/10× EAB medium yielded lower biomass at 163.8 ± 9.1 mg L^-1^ and lower growth rate at 1.13 ± 0.01 day^-1^ compared to 201.9 ± 9.3 mg L^-1^ and 2.71 ± 0.06 day^-1^ generated by microalgae grown in the 1× EAB medium. Though, the EAB can be considered as *E. coli* eﬄuents, these results indicated that initial EAB was not inhibitory against the algal growth. The cell density and the biomass in medium TAP were much higher as compared to the spent bacterial media (Supplementary Figure 1), which may be due to the different concentrations of acetate available in mediums used (1 mg/mL in TAP medium and 0.5 mg/mL in the spent bacterial media). We conclude that these low levels of acetate in the media perhaps were not sufficient to support a long exponential growth phase of algae.

**Table 1:** Growth parameters of *C. reinhardtii* cultivated in EAB under mixotrophic and heterotrophic conditions.

	Mixotrophic growth				Heterotrophic growth			
	1×	1/2×	1/5×	1/10×	1×	1/2×	1/5×	1/10×
Biomass production (mg/L)	201.9 ± 9.3	184.2 ± 8.4	175.5 ± 8.2	163.8 ± 9.1	45.6 ± 2.3	29.4 ± 1.5	15.8 ± 0.9	12.1 ± 0.9
Specific growth rate (day^-1^)	2.71 ± 0.06	2.68 ± 0.05	1.45 ± 0.05	1.13 ± 0.01	1.80 ± 0.02	1.86 ± 0.03	1.75 ± 0.02	1.02 ± 0.01

*Data are represented as the means ± SD (*n* = 3).*

**Figure 2B** showed the time course growth profiles of *C. reinhardtii* cultivated under heterotrophic conditions at the four nutrient concentration levels. *C. reinhardtii* grown in 1/10× EAB had the lowest growth rate and the biomass production was only 12.1 ± 0.9 mg/L. Except culture in 1/10× EAB, the other cultures possessed similar growth rate (around 1.80 day^-1^) and different duration of exponential phase, which caused the different biomass production that were 45.6 ± 2.3, 29.4 ± 1.5, and 15.8 ± 0.9 mg/L in 1×, 1/2×, and 1/5× EAB, respectively (**Table 1**). These results suggested that the acetate ranging in a certain concentration could not affect the specific growth rate but extend the exponential phase under heterotrophic conditions. Consequently, the final biomass production was related to the initial acetate concentration.

Till date, according to best of our knowledge, acetate is the only organic carbon source supporting *C. reinhardtii* growth. It seemed that the acetate consumption rates were similar at the same dilution ratio of EAB under two conditions (**Figure 3**). However, the biomass production under heterotrophic cultivation was lower than that obtained under the mixotrophic conditions at the same dilution ratio of EAB. From our results, it is most likely that the initial acetate and the illumination simultaneously stimulate the optimum growth up.

### Nutrients Removal of EAB

**FIGURE 4:**
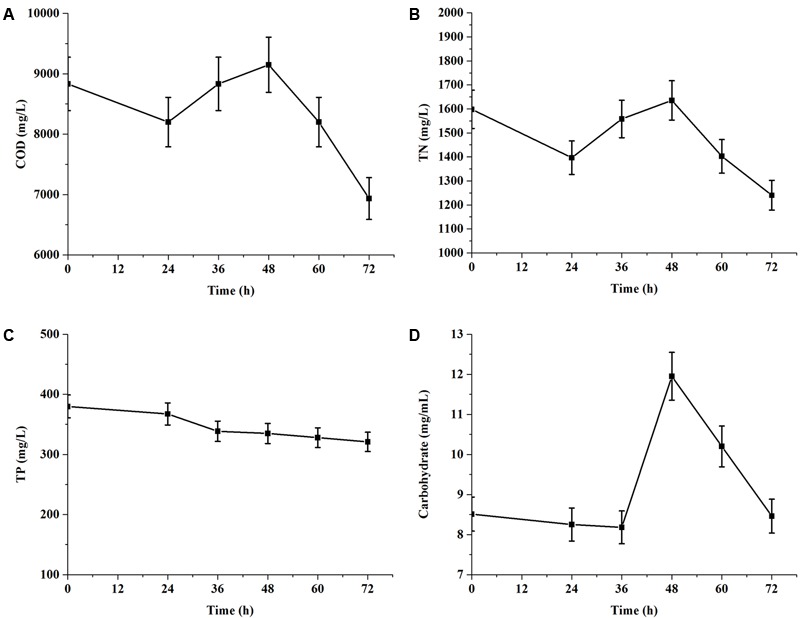
Chemical oxygen demand and nutrients removal of *C. reinhardtii* grown in 1× EAB under mixotrophic condition. Data are represented as the means ± SD (*n* = 3). (**A**, Time course of COD; **B**, Time course of total nitrogen; **C**, Time course of total phosphorus; **D**: Time course of carbohydrate).

According to above results, the experiments of nutrient removal were conducted in 1× EAB and the results were shown in **Figure 4**. During 72 h of cultivation, the consumption rates of COD and TN were 21.51 and 22.41%, respectively. However, COD (**Figure 4A**) value re-increased after a decrease within 24 h and further continued to re-decreased dramatically after 48 h. TN concentration curve (**Figure 4B**) paralleled the trend line of COD value and carbohydrate content (**Figure 4D**) also re-increased significantly from 36 to 48 h. These data indicated that *C. reinhardtii* could secret extracellular organic matters (EOM) during exponential phase. And these EOM consisted mainly of nitrogen metabolite and some carbohydrates. These results were consistent with Vandamme et al. (2012) reported that microalgae can release significant amount of organic matters, which may potentially increase the COD level of wastewater. Phosphorus is an important nutrient element for microalgae. The removal of TP was only 15.53% from initial EAB containing 379.88 mg/L in 3 days. The terminal value of phosphorus concentrations in culture medium was still high (**Figure 4C**), compared with the terminal value of TN. It implied that TP in the medium was not the limiting nutrient for *C. reinhardtii* growth.

### Carbohydrate and Lipid Accumulation during EAB Nutrients Removal

**FIGURE 5:**
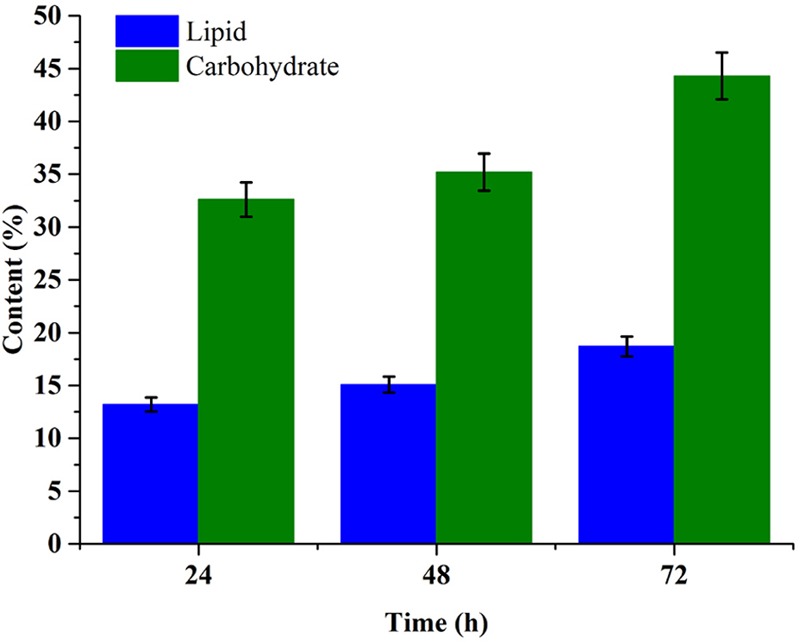
Time course profile of the lipid and carbohydrate content of *C. reinhardtii* biomass obtained from mixotrophic cultivation in 1× EAB. Data are represented as the means ± SD (*n* = 3).

Carbohydrate and lipid accumulation is related closely with carbon resource metabolism in microalgae cell. **Figure 5** showed the carbohydrate and lipid accumulation trend in *C. reinhardtii* under mixotrophic cultivation in 1× EAB. The carbohydrate content increased from 35.2% at 48th hour (exponential phase) to 44.3% at 72nd hour (stationary phase). The significant increment was consistent with the fact that intracellular carbohydrate accumulation often takes place at later growth stage. The trend of lipid accumulation was similar with the one of carbohydrate accumulation. However, the final content of lipid was 18.7% that was lower than previous reports due to the relative higher nitrogen and phosphorus concentration in 1× EAB (Iwai et al., 2015; Silva and Sforza, 2016). Besides lipid, this microalgae cells contained abundant carbohydrate, which indicated the potentialities of the algal biomass as ideal feedstock for both biofuel and other chemicals production.

### Fatty Acid Composition of Lipid

Fatty acid composition depends on the environmental factors and medium composition and it has significant effect on feedstock quality directly to produce biodiesel. **Table 2** listed the fatty acid composition of lipid accumulated by *C. reinhardtii* under mixotrophic conditions. Palmitic acid (C16:0) and linolenic acid (C18:3) were predominant in the whole culturing period, although there was an increment in the level of oleic acid (C18:1) in the later phase. Small content of fatty acid: myristic acid (C14:0), palmitoleic acid (C16:1) and stearic acid (C18:0) were also detected in the later phase. The ratio of saturated and unsaturated fatty acids was 67.4:32.6 at the exponential phase and it varied slightly (67.2:32.8) at the end of the culture period though the fatty acid composition changed significantly. These data suggested that the composition of algal lipids was largely regulated by physiological status. Additionally, linolenic acid (C18:3) should be lower than 12% for a quality biodiesel according to European Standard EN14214. In order to be consistent with this standard, the algal growth conditions should be further improved.

**Table 2:** Fatty acids composition of lipids accumulated by *C. reinhardtii* cultivated in 1× EAB under mixotrophic conditions.

Time (h)	C14:0	C16:0	C16:1	C18:0	C18:1	C18:2	C18:3
24	–	67.4	–	–	–	–	32.6
48	3.3	61.0	4.0	5.4	14.9	–	11.4
72	3.8	57.9	4.6	5.5	14.4	–	13.8

*Data are represented as the means ± SD (*n* = 3).*

## Discussion

The primary aim of our study was to develop novel method to produce an economically and environmentally sustainable feedstock from algae grown on nutrients from EAB. EAB contained acetic acid and other residual nutrients, the original EAB could be utilized by a green microalgal strain *C. reinhardtii* directly to produce biomass and remove 21.51% COD, 22.41% TN and 15.53% TP without inhibition. The algal biomass consisted of 18.7% lipid and 44.3% polysaccharides. Thus, this microalgal strain has the potential to grow in EAB, and to remove nutrients efficiently and produce the microalgal biomass as well.

Microalgae are known to utilize nutrients from organic-rich agricultural (Wang et al., 2012), industrial (Roberts et al., 2013) and municipal wastewater (Wang et al., 2010) to remediate excess nutrients from the eﬄuents. In the past years, several reports illustrated the growth of microalgae for industrial applications but due to different experimental conditions and calculation methodologies adopted in different studies, comparisons between them cannot be made accurately. Moreover, the results of biomass accumulation and nutrients removal efficiency correspond to microalgae species.

*Chlamydomonas reinhardtii* is a model species of green algae; several studies anticipate that it could be an ideal strain to treat the spent media in this study. Previous studies also concluded that *Chlamydomonas* sp. can utilize industrial waste effectively as well as it can achieve the maximum biomass growth rate to produce biodiesel or biofuels (Wu et al., 2012).

On the other hand, Wang et al. (2012) previously reported that culturing microalgae in mixotrophic mode with diluted primary piggery wastewater can be an effective way for conversion of high-strength livestock wastewater to a profitable byproduct as well as minimizing the contamination of the environment. But, nutrient-rich raw piggery wastewater also carries several disadvantages, including high concentrations of urea, ammonia, organic carbons and pesticides, high turbidity and unbalanced C:N:P ratios, which ultimately affect the growth of algae. Qi et al. (2017), reported the culturing of *C. reinhardtii* in diluted primary piggery wastewater at various CO_2_ concentrations. These authors summarize that highest removal efficiency of COD was 42.0% with 5% CO_2_ aeration on the 2nd day followed by no further COD reduction in the last 4 days. Sforza et al. (2012) proposed that excess CO_2_ could restrain the heterotrophic metabolic pathway of the organic substrate, though photosynthesis was stimulated in *Nannochloropsis salina* and *Chlorella protothecoides*. Another study also reported the removal efficiency of the organic carbon substrate declined in the group with 15% CO_2_ supply (Qi et al., 2017).

Mostly organic waste streams harbor several bacteria which can stimulate or inhibit microalgal growth. In these symbiotic environments, the microalgae can supply oxygen and extracellular compounds to heterotrophic bacteria and heterotrophic bacteria can supply CO_2_, low molecular weight organic substances, and vitamin B_12_ to the photosynthetic partner in return. However, some bacteria can lead to lyses of microalgae cells which ultimately results into the microalgal death. On the other hand, recent studies have adapted the artificial co-cultivation of a single microalgal strain with each growth-promoting bacterium or co-cultivation with bacteria present naturally in wastewaters to enhance the microalgal biomass productivity and nutrient removal (Cho et al., 2017).

Our study demonstrated that mixotrophic culture conditions using acetate can enhance microalgal biomass and lipid productivities together with nutrient removal by a single strain of microalgae. Moreover, *Chlamydomonas* can grow in EAB and remove the COD, P and N from the medium. The main reason is that the *E. coli* can release some acetate which can be utilized by *Chlamydomonas*. Glucose cannot support the growth of *C. reinhardtii.* Whereas, acetate is the only organic carbon source supporting *C. reinhardtii* growth which is in consistency with the previous report by Ding et al. (2016) (Supplementary Figure 2). Until recently, organic carbon substrates have only been studied as single substrate to sustain microalgae growth in heterotrophic conditions. Nevertheless, in carbon-rich wastewaters or industrial eﬄuents, several carbon sources are usually available to support the microalgae growth (Lowrey et al., 2015; Turon et al., 2016).

Although the growth of *C. reinhardtii* was clearly increased by mixotrophic cultivation, use of pure acetate as a carbon source would be a very expensive means of biofuel production. Therefore, we investigated the use of EAB harboring acetate as an alternative carbon source for mixotrophic cultivation.

To our knowledge, the present work constitutes to demonstrate that *C. reinhardtii* can be grown mixotrophically with eﬄuent harboring acetate as a carbon source. Our results clearly indicate that mixotrophic cultivation of *C. reinhardtii* using acetate provides better performance in terms of algal biomass and lipid production. At the same time, *C. reinhardtii* is not suitable for heterotrophic cultivation under the diverse conditions that we tested. Acetate was the most effective carbon source in terms of algal growth and lipid biosynthesis indicating that eﬄuent harboring acetate is less expensive and cost-effective alternative carbon source (Moon et al., 2013). To our knowledge, the present work constitutes the first study to demonstrate that *C. reinhardtii* can be grown mixotrophically in EAB. EAB contain significant levels of acetic acid, which is likely the most significant carbon source (Supplementary Table 1).

Additionally, most COD, TN and TP could not be removed in this study. As known, nitrogen and phosphorus serve as the most essential elements for microalgae assimilation, growth and energy metabolism, respectively. Ding et al. (2016) have reported the phosphorus removal efficiency from 38.15 to 68.53% with different ratios of eﬄuents. One recent study also reported that algae could not remove nitrogen from wastewater sufficiently (Álvarez-Díaz et al., 2017). Kong et al. (2010) concluded that the main reason for less removal of N and P loadings could be the lower growth rate or other parameters such as N/P ratio may not be optimized for the best performance of *C. reinhardtii* in terms of N and P removal in the Erlenmeyer flasks compared with the large-scale bioreactor. The complete assimilation of P by all species could be related to the phosphorus luxury uptake process (Powell et al., 2009). The phosphorus storage mechanism that occurs in naturally through which microalgae can be phosphorus-starved for a period; therefore, algae can accumulate higher amounts of phosphorus than needed. Whereas, P loss through precipitation due to high pH is discarded due to the continuous CO_2_ injection that maintained low pH values in all reactors which led to removal of only 12–24% of dissolved phosphorus. In present study, the removal amounts of COD, TN and TP were 1900, 350, and 60 mg/L around, respectively. These values were not as lower. There are studies which depict the comparable range of COD, TN and TP removal in waste water treatment (de la Noüe et al., 1992).

Accordingly, EAB can possibly support the microalgal biomass production due to sufficient nutrients content. Therefore, cultivation of algae in EAB has been proved to be potentially practical and economical strategy for algal feedstock production and organic wastewater treatment. However, the strategy of EAB utilization should be studied further in order to optimize the biomass growth and nutrients removal in a better dimension for achieving high growth rates of microalgae.

## Author Contributions

Z-JW designed the study, wrote the protocol. J-GZ conducted all experiments. FZ performed the data analysis. KT took part in the manuscript preparation. FH took part in part experiment and analyzed the data. All authors read and approved the final manuscript.

## Conflict of Interest Statement

The authors declare that the research was conducted in the absence of any commercial or financial relationships that could be construed as a potential conflict of interest. The reviewer KH and handling Editor declared their shared affiliation, and the handling Editor states that the process nevertheless met the standards of a fair and objective review.

## Footnotes

1 http://chlamycollection.org/
