# Supplementary material for: Valorization of Spent Escherichia coli Media Using Green Microalgae Chlamydomonas reinhardtii and Feedstock Production
Source: Front Microbiol. 2017 Jun 7;8:1026. doi: 10.3389/fmicb.2017.01026 (PMC5461289; doi:10.3389/fmicb.2017.01026)
Supplement: Supplementary file 1 [file Data_Sheet_1.DOCX]

**Supplemental Figure1.**The growth curve of *C. reinhardtii* in TAP medium. Bimass(mg/L)=OD_680_×0.3893×1000. Data are represented as the means ±SD (n=3).


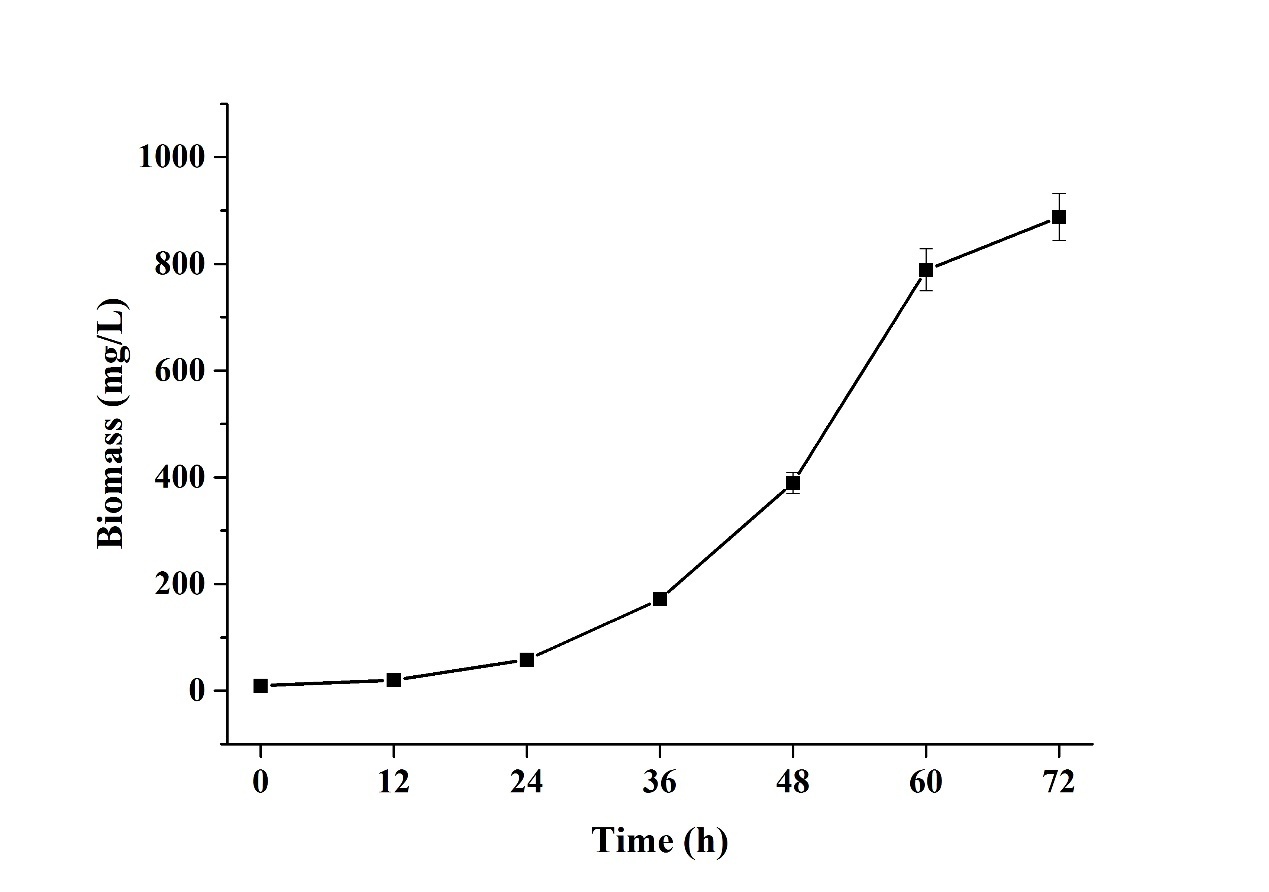


**Supplemental Figure2.** The effect of carbon sources on the growth of *C. reinhardtii* under mixotrophic condition at 0.1% of carbon source, whereas the other compounds were same as TAP medium.


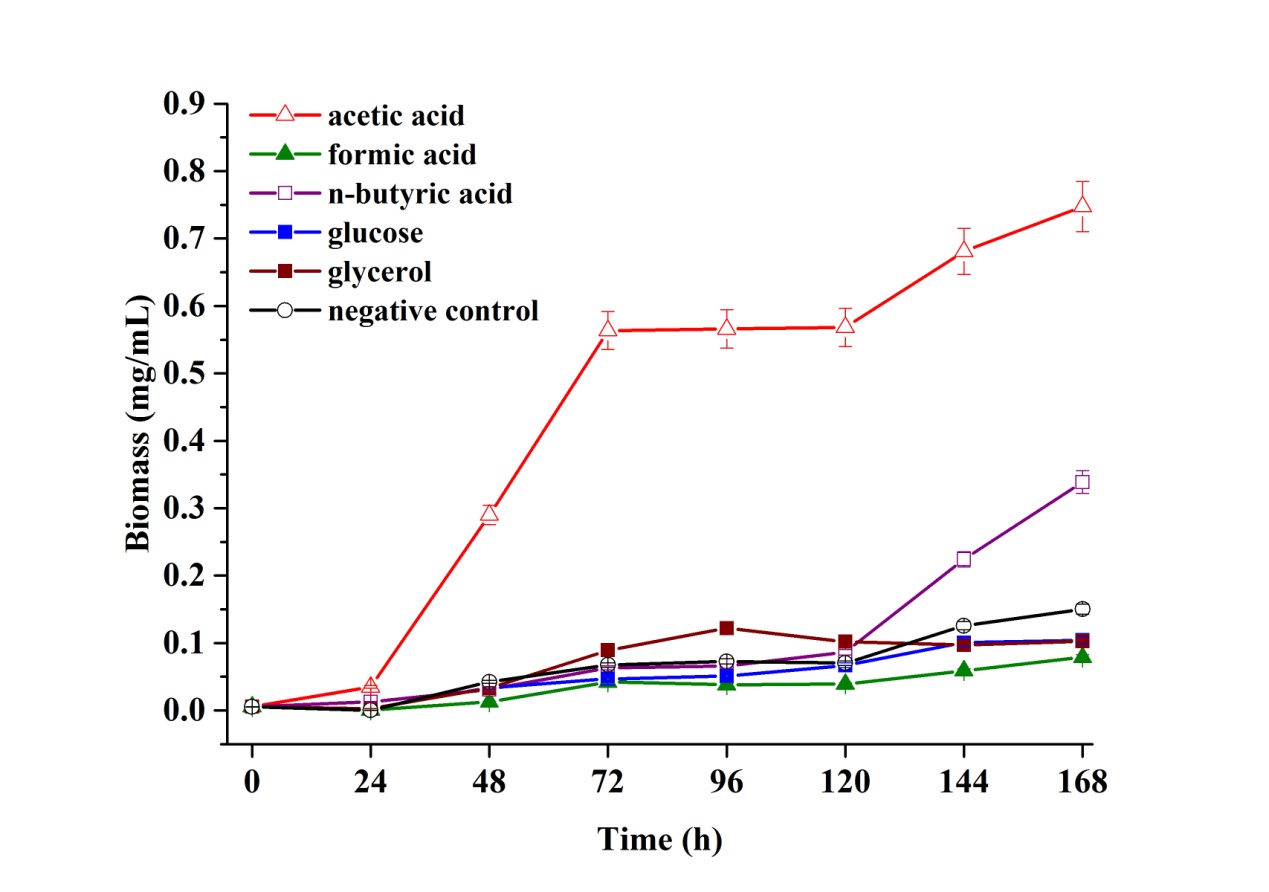


**Supplement Table 1.**

**Comparison of present strategy (EAB) medium with previous wastewater treatments for culturing microalgae**

| **Advantage** | **Disadvantage** |
| --- | --- |
| EAB | Previous strategies |
| **1.** Our study depicts that *C. reinhardtii* is easy to culture. Our strategy is cost effective and environmental friendly.  **2.** We have targeted the reutilization of spent medium to use C, N and P for culturing microalgae.  **3.** Microalgae can easily convert acetate into acetyl-CoA which is the main precursor for carbon compounds synthesis (Ramanan et al., 2013). Thus, coupling of the effluents treatment to microalgae production can not only decrease the feedstock’s cost but also benefit environment protection (Lau et al., 1996).  **4.** Acetate can enhance microalgal biomass and lipid productivities together. Whereas, Glucose cannot support the growth of *C. reinhardtii.* Acetate is the only organic carbon source supporting *C. reinhardtii* growth which is in consistency with the previous report by Ding et al. (2016).  To summarize, the importance of our strategy was the available carbon substrate (acetate) for microalgae growth because *E.coli* could release sufficient amount of acetate which was further utilized by *Chlamydomonas*. | **1.** Nutrient-rich raw piggery and municipal wastewater have high concentrations of urea, ammonia, organic carbons and pesticides, high turbidity and unbalanced C:N:P ratios, which affect the growth of algae (Wang et al. 2012).  **2.** The waste water medium contains many complex nutrients which are not readily available for uptake by microalgae and increase the lag phase.  **3.** Most organic waste streams harbor numerous bacteria that can stimulate or inhibit microalgal growth. These bacteria can lyse microalgal cells by direct contact or by indirect contact mediated by extracellular substances that they excrete, resulting in the death of microalgae. Therefore, in a mixed culture of microalgae and bacteria, the relationship between the groups is suggested to be complicated.（Qi et al., 2017） |
